# Global Scientific Research Landscape on Medical Informatics From 2011 to 2020: Bibliometric Analysis

**DOI:** 10.2196/33842

**Published:** 2022-04-21

**Authors:** Xuefei He, Cheng Peng, Yingxin Xu, Ye Zhang, Zhongqing Wang

**Affiliations:** 1 Department of Ophthalmology Hwa Mei Hospital University of Chinese Academy of Sciences Ningbo China; 2 Department of Ophthalmology The Fourth Affiliated Hospital of China Medical University Shenyang China; 3 Information Center The First Hospital of China Medical University Shenyang China

**Keywords:** medical informatics, bibliometrics, VOSviewer, data visualization

## Abstract

**Background:**

With the emerging information and communication technology, the field of medical informatics has dramatically evolved in health care and medicine. Thus, it is crucial to explore the global scientific research landscape on medical informatics.

**Objective:**

This study aims to present a visual form to clarify the overall scientific research trends of medical informatics in the past decade.

**Methods:**

A bibliometric analysis of data retrieved and extracted from the Web of Science Core Collection (WoSCC) database was performed to analyze global scientific research trends on medical informatics, including publication year, journals, authors, institutions, countries/regions, references, and keywords, from January 1, 2011, to December 31, 2020.

**Results:**

The data set recorded 34,742 articles related to medical informatics from WoSCC between 2011 and 2020. The annual global publications increased by 193.86% from 1987 in 2011 to 5839 in 2020. Journal of Medical Internet Research (3600 publications and 63,932 citations) was the most productive and most highly cited journal in the field of medical informatics. David W Bates (99 publications), Harvard University (1161 publications), and the United States (12,927 publications) were the most productive author, institution, and country, respectively. The co-occurrence cluster analysis of high-frequency author keywords formed 4 clusters: (1) artificial intelligence in health care and medicine; (2) mobile health; (3) implementation and evaluation of electronic health records; (4) medical informatics technology application in public health. COVID-19, which ranked third in 2020, was the emerging theme of medical informatics.

**Conclusions:**

We summarize the recent advances in medical informatics in the past decade and shed light on their publication trends, influential journals, global collaboration patterns, basic knowledge, research hotspots, and theme evolution through bibliometric analysis and visualization maps. These findings will accurately and quickly grasp the research trends and provide valuable guidance for future medical informatics research.

## Introduction

### Background

The field of medical informatics is dedicated to systematically processing data, information, and knowledge in medicine and health care [1]. In the 1950s, Robert S Ledley and Lee Browning Lusted first performed the complicated reasoning processes inherent in medical diagnosis using electronic computers to minimize medical errors primarily [2]. Given that the emerging information and communication technology is being continuously applied in the medical field, medical informatics, as a discipline, has dramatically evolved over the past 70 years and brought about significant changes to human social needs [2,3]. Recent advances in health care information technology, electronic health records (EHRs), health data standards, and health information exchange have become the major focus of scientific research [4]. Therefore, health informatics, which represents the development of systems and methods for acquisition, processing, handling, communication, storage, retrieval, management, discovery, analyzing, and synthesizing patient information to improve health and health care, is more often used in the literature [5-8]. The number of publications and journals focusing on medical informatics/health informatics has multiplied in recent years. Therefore, it is essential to explore the global scientific research landscape in this discipline.

Bibliometrics is defined as scientific and quantitative research of publications, which describes the research trends of a certain research field using statistical methods to analyze a large number of publications [9]. In 2007, Bansard et al [10] first presented a bibliometric study on medical informatics and bioinformatics, which mainly identified the present links and potential synergies between the bioinformatics and medical informatics research areas. Subsequently, bibliometric analyses on specific medical informatics technology have been performed, such as those on mobile health research [11], shared decision making [12], telemedicine [13-15], computer-aided diagnosis [16], natural language processing [17], artificial intelligence (AI) in health care [18], digital health [19,20], among others.

### Objective

This study aims to analyze medical informatics as a discipline (a catalog from the Web of Science Core Collection [WoSCC]) and demonstrate the longitudinal trends from the global perspective. Thus, we performed bibliometric analysis and prepared visualization maps to identify and present the publication trends, global collaboration patterns, basic knowledge, research hotspots, and emerging hotspots in medical informatics.

## Methods

### Data Collection

WoSCC is the most widely used database in various scientific fields, including over 9000 high-level academic journals worldwide. The research area is generated by a set of classification methods for all databases under WoSCC. Therefore, documents of the same research area or discipline can be identified, retrieved, and analyzed from WoSCC for bibliometrics analysis [21]. Medical informatics is one of the 252 research areas of WoSCC. For search purposes, the retrieval research area was set as “medical informatics”, the period was set as “from 2011 to 2020”, the document type was set as “article”, and the language was set as “English”. We conducted our search strategy in WoSCC on June 1, 2021, at China Medical University.

We identified and incorporated 34,742 studies on medical informatics from WoSCC. The full record and cited references of the retrieved publications were collected and saved in text formats (eg, .txt). The data used in this study are publicly available and associated with no protected health information.

### Ethics Consideration

Publicly available data were searched and downloaded from WoSSC. The extraction of this data did not involve interaction with human subjects or animals. Thus, there were no ethical issues involving the use of these data, and no approval from an Ethics Committee was required.

### Analytical Tool and Visualization Maps

The most commonly used bibliometric methods are co-authorship, co-citation, and co-occurrence. Co-authorship analysis reveals collaboration patterns among authors, institutions, and countries [22]. Co-citation analysis contributes to discovering and determining the knowledge base of one discipline [23]. Co-occurrence analysis uses the frequency of multiple words in the same article to identify how close they are, thereby helping researchers identify hot topics and trends in the discipline. VOSviewer [24] is an excellent bibliometric analysis software developed by van Eck and Waltman [25,26]. It calculates the similarity *s_ij_* of 2 items *i* and *j* with the equation *s_ij_* = *c_ij_*/*(w_i_w_j_)*, where *c_ij_* denotes the number of co-occurrences of items *i* and *j*, and *w_i_* and *w_j_* denote the total number of occurrences of items *i* and *j*. Once the similarity matrix is created, VOSviewer maps all the items in a 2D map so that items with a high similarity will be located close to each other, while those with a low similarity will be located far from each other. In this study, we employed VOSviewer version 1.6.16 to extract bibliometric information such as publication year, journals, authors, institutions, countries/regions, references, and keywords. Besides, we employed VOSviewer to conduct co-authorship analysis, co-citation analysis, co-occurrence analysis, and then built visualization network maps.

## Results

### Global Publications on Medical Informatics

A total of 34,742 articles on medical informatics were retrieved. The average annual number of publications was 3474 during the past decade. The annual global publications on entire life sciences and biomedical sciences are presented in Multimedia Appendix 1. The annual global publications on entire life sciences and biomedical sciences increased 55.73% from 573,981 to 893,887 from 2011 to 2020. The annual global publications on medical informatics increased 193.86% from 1987 to 5839 from 2011 to 2020, which was the highest increase rate in the life sciences and biomedical fields.

**Figure 1:**
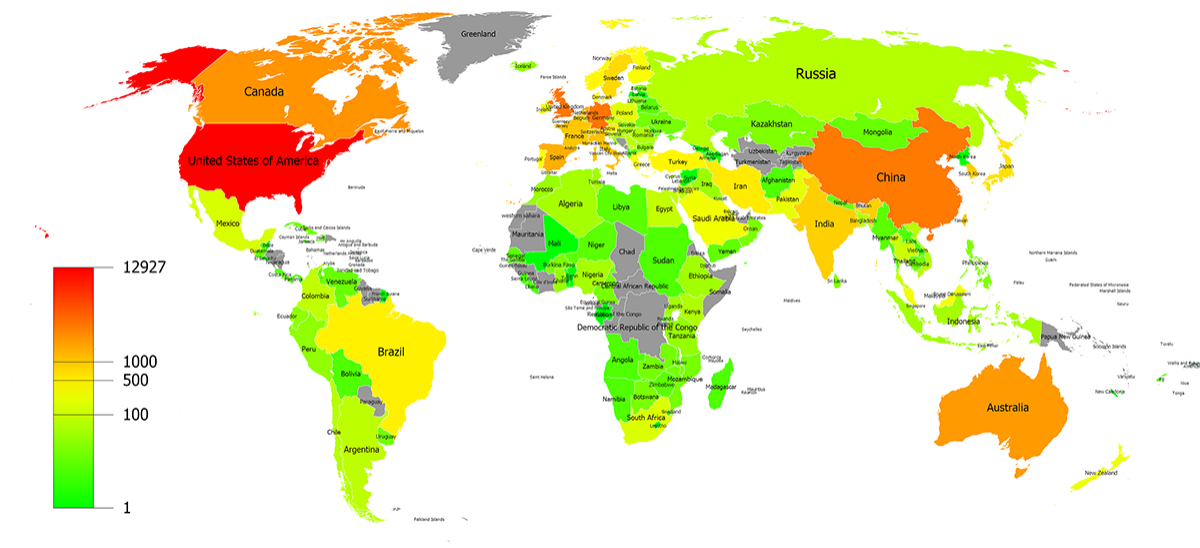
The global distribution of countries/regions participating in medical informatics research from 2011 to 2020.

**Figure 2:**
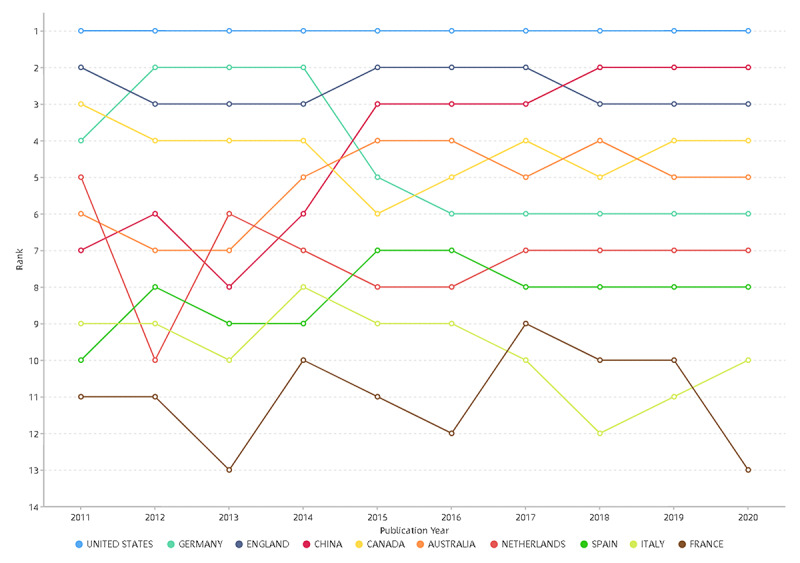
The annual ranking changes of the top 10 most productive countries regarding publication of articles on medical informatics from 2011 to 2020.

The global distribution of countries/regions participating in medical informatics research is shown in Figure 1. A total of 161 countries/regions contributed to medical informatics from 2011 to 2020. The top 10 countries contributed 27,213 articles in medical informatics. The United States (12,927 publications) is the most productive country, followed by Germany (3336 publications), England (3269 publications), China (3157 publications), and Canada (2237 publications). Changes in the annual ranking of the top 10 most productive countries for medical informatics research are shown in Figure 2. The rankings of the top 10 countries changed every year from 2011 to 2020, but the United States consistently ranked first in publications.

### Contribution of Source Journals

Based on the retrieved results, articles on medical informatics were distributed in 37 journals. The top 10 journals with the most publications in the medical informatics discipline are presented in Table 1. From 2011 to 2020, *Journal of Medical Internet Research* with 3600/34,742 (10.36%) publications was the top productive journal, followed by *Statistics in Medicine* (3282 publications) and *Computer Methods and Programs in Biomedicine* (2409 publications). *Journal of Medical Internet Research* with 63,932 citations was also the most highly cited journal, followed by *Statistics in Medicine* (45,042 citations) and *Journal of the American Medical Informatics Association* (36,874 citations).

**Table 1:** The top 10 most productive journals in Medical Informatics from 2011 to 2020.

Rank	Journal	Country	Publication start year	Total publications	Total citations
1	*Journal of Medical Internet Research*	Canada	1999	3600	63,932
2	*Statistics in Medicine*	England	1982	3282	45,042
3	*Computer Methods and Programs in Biomedicine*	Ireland	1985	2409	35,157
4	*Biomedical Engineering-Biomedizinische Technik*	Germany	1971	2184	3541
5	*Journal of Medical Systems*	United States	1977	2104	28,747
6	*Journal of The American Medical Informatics Association*	England	1994	1746	36,874
7	*Journal of Evaluation in Clinical Practice*	England	1995	1676	13,720
8	*BMC Medical Informatics and Decision Making*	England	2001	1660	17,956
9	*IEEE Journal of Biomedical and Health Informatics*	United States	2013	1656	27,850
10	*JMIR mHealth And uHealth*	Canada	2013	1544	16,156

### Contributions of Authors and Institutes

A total of 114,841 authors (175,530 frequency) contributed to medical informatics from 2011 to 2020. As shown in Table 2, David W Bates (99 publications) was the most productive author, followed by Hua Xu (73 publications), and George Hripcsak (61 publications). Ian R White (5928 citations) was the most cited author, followed by David W Bates (2019 citations) and Joshua C Denny (1924 citations).

A total of 20,513 institutions contributed to the medical informatics field from 2011 to 2020. The number of institutions that issued more than 10 publications was 1385. Harvard University (1161 publications) was the most productive institution, followed by University of Toronto (503 publications), University of Washington (488 publications), and Columbia University (462 publications).

**Table 2:** The top 10 productive journals, authors, and institutions of medical informatics.

Rank	Author	Total publications	Total citations	Institution	Total publications	Total citations
1	David W Bates	99	2019	Harvard University	1161	19,471
2	Hua Xu	73	1691	University Toronto	503	10,904
3	George Hripcsak	61	1465	University Washington	488	6994
4	Dean F Sittig	61	1233	Columbia University	462	7744
5	Adam Wright	59	1102	University Michigan	455	6737
6	Hongfang Liu	58	873	Stanford University	422	7976
7	Joshua C Denny	51	1924	Vanderbilt University	389	8971
8	Chunhua Weng	51	818	University Penn	357	4084
9	Xiaoqian Jiang	48	565	Duke University	336	5375
10	Ian R White	47	5928	Mayo Clinic	326	5066

### Co-authorship Analysis of Authors, Institutions, and Countries

Upon analyzing the 34,742 retrieved articles, there was an average of 5 co-authors for each article, revealing extensive co-authorships among authors in the field of medical informatics. We employed VOSviewer to analyze the co-authorship of authors, institutions, and countries/regions and then built the visualization network map.

**Figure 3:**
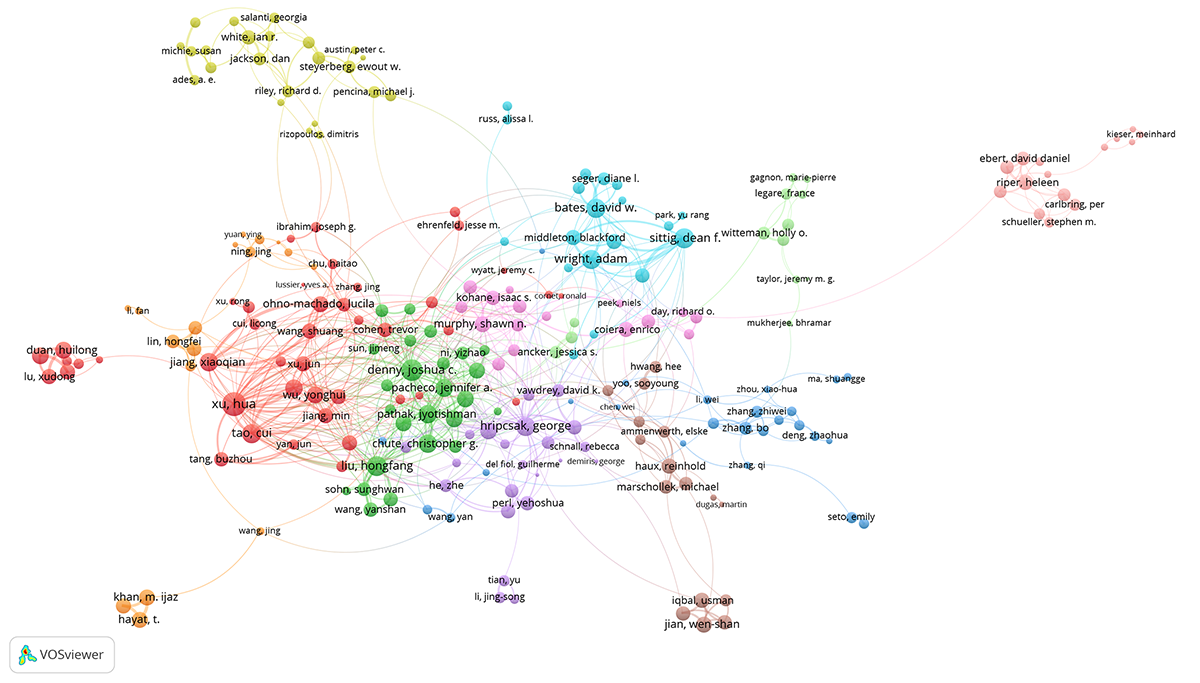
The collaborative network of productive authors participating in medical informatics publications from 2011 to 2020.

**Figure 4:**
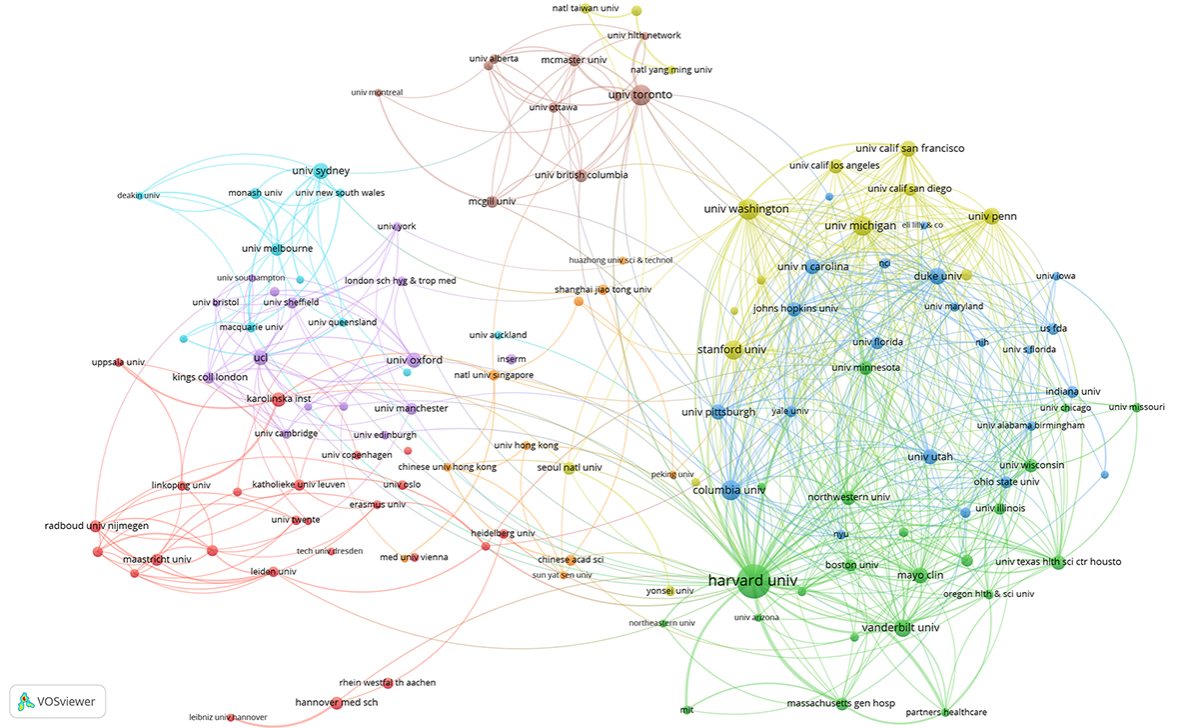
The collaborative network of productive institutions participating in medical informatics publications from 2011 to 2020.

**Figure 5:**
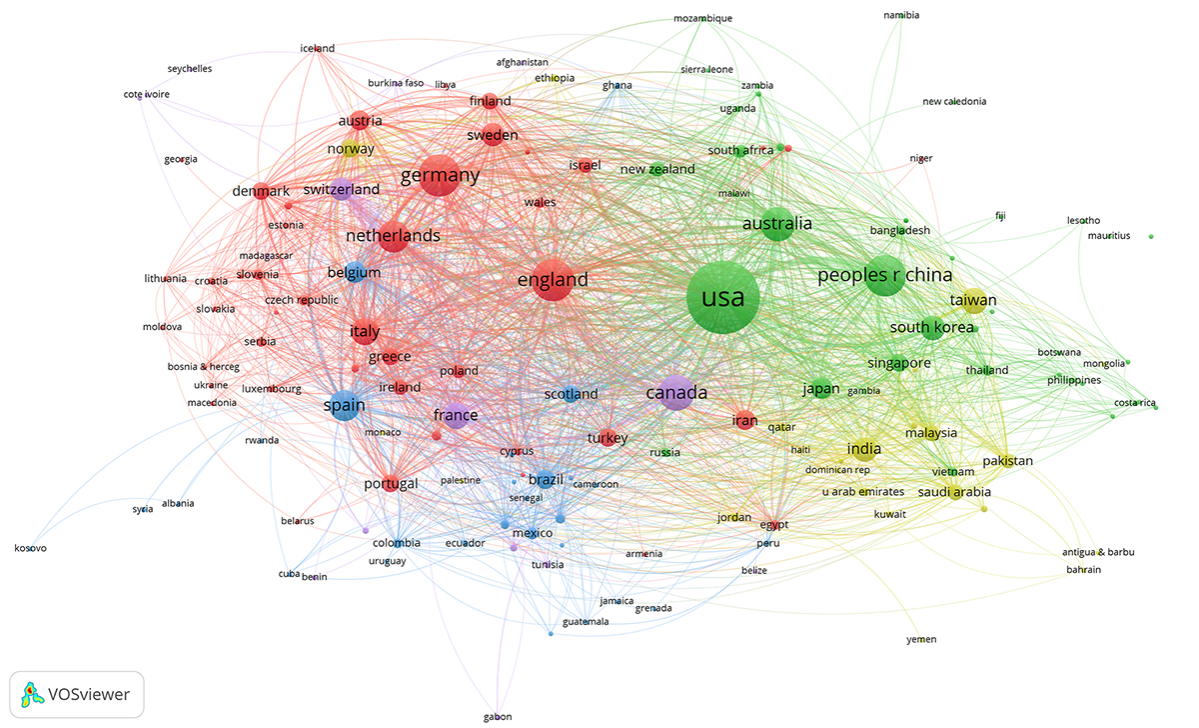
The collaborative network of countries/regions participating in medical informatics publications from 2011 to 2020.

We found that 304 productive authors published more than 15 articles. As shown in Figure 3, the largest collaborative network of productive authors comprising 234 authors was divided into 11 clusters of different colors. Hua Xu was the most active co-author with a total link strength of 162. The largest cluster (in red) involved 43 co-authors centering on Hua Xu, Xiaoqian Jiang, and Cui Tao. Figure 4 shows the collaborative network of 133 productive institutions that published more than 100 articles by 8 clusters of different colors. Harvard University was the most active co-author institution with a total link strength of 1484 and in the center of the green cluster. Figure 5 shows the largest collaborative network of countries/regions comprising 158 countries/regions divided into 4 different colored clusters. The United States was the most co-author country with a total link strength of 5495.

### Co-citation Analysis of References and Cited Journal

**Figure 6:**
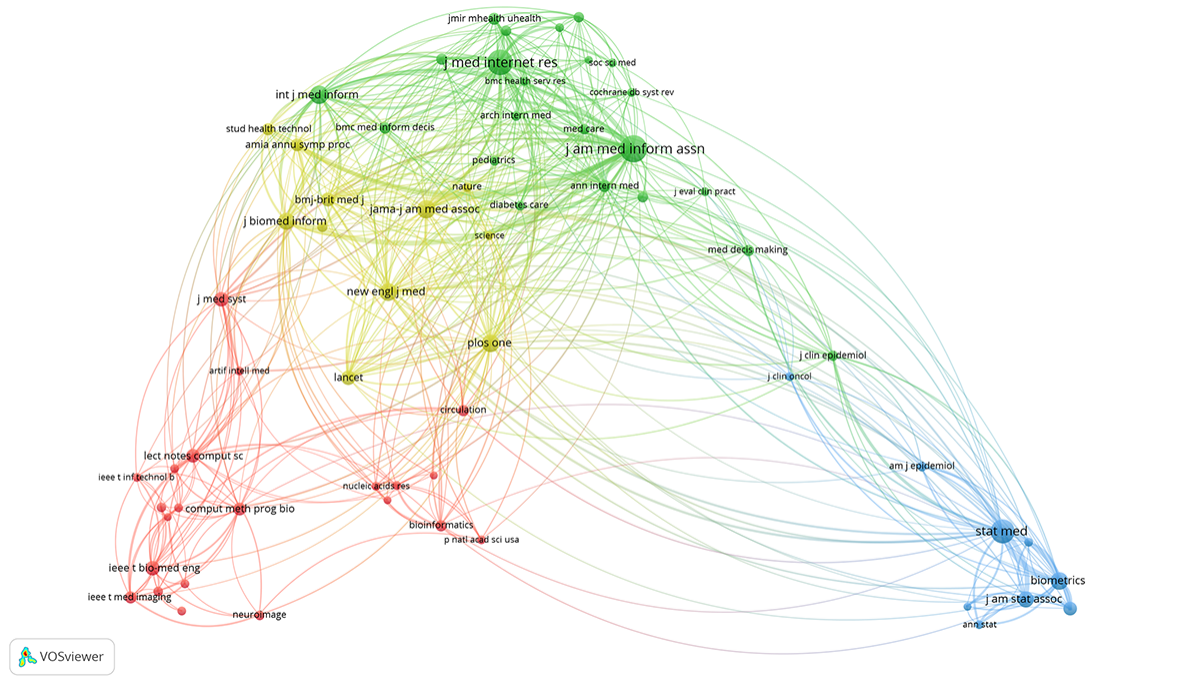
The co-citation network of highly cited journals on medical informatics from 2011 to 2020.

A total of 34,742 retrieved articles cited 700,628 references from 157,424 journals. The article published by Breiman in 2001 entitled “random forests” was the most cited reference. This article was cited 567 times in the retrieved publications on medical informatics and 40,253 times in WoSCC. Furthermore, the analysis of the distribution of cited journals helps identify the knowledge base of a certain field. The co-citation network of 64 cited journals with a minimum of 2000 citations was divided into 4 clusters of different colors. As shown in Figure 6, the red cluster centered on *IEEE Transactions on Biomedical Engineering,* and *IEEE Transactions on Medical Imaging, Computer Methods and Programs in Biomedicine*; the green cluster centered on *Journal of Medical Internet Research*, *Journal of the American Medical Informatics Association,* and *International Journal of Medical Informatics*; the blue cluster centered on *Statistics in Medicine, Biometrics, Journal of The American Statistical Association*; and the yellow cluster centered on *JAMA, New England Journal of Medicine, Lancet,* and *PLoS One.*

### Co-occurrence Analysis of Author Keywords

**Figure 7:**
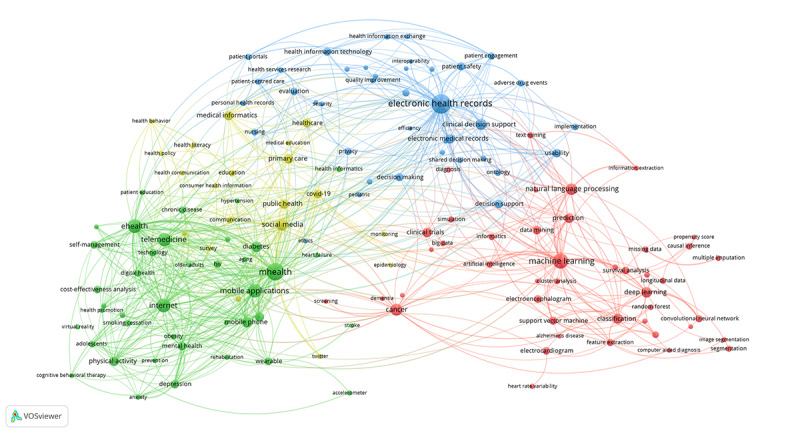
The co-occurrence network of high-frequency author keywords on medical informatics publications from 2011 to 2020.

The primary purpose of keywords is to provide fast access to scientific publications for researchers. In a bibliometric study, co-occurrence analysis of keywords effectively reflects the research hotspots in a scientific field [27,28]. This study analyzed the “author keywords” retrieved from WoSCC to represent the research hotspots. We employed VOSviewer to perform a co-occurrence analysis of the 143 high-frequency author keywords, which appeared more than 100 times from 2011 to 2020. The co-occurrence network map of high-frequency author keywords on medical informatics is shown in Figure 7. The most high-frequency author keyword was *EHRs* (with 1591 occurrences), followed by *mHealth* (n=1331), *machine learning* (n=994), *internet* (n=827), and *eHealth* (n=824). The 143 high-frequency author keywords formed 4 clusters: red, green, blue, and yellow. The red cluster is the largest one with 43 keywords regarding the research hotspots of AI in health care and medicine. The green cluster mainly focused on the research hotspots of mobile health; the blue cluster represented the research hotspots of implementation and evaluation of EHRs; the yellow cluster demonstrated the research hotspots of medical informatics technology application in public health.

**Figure 8:**
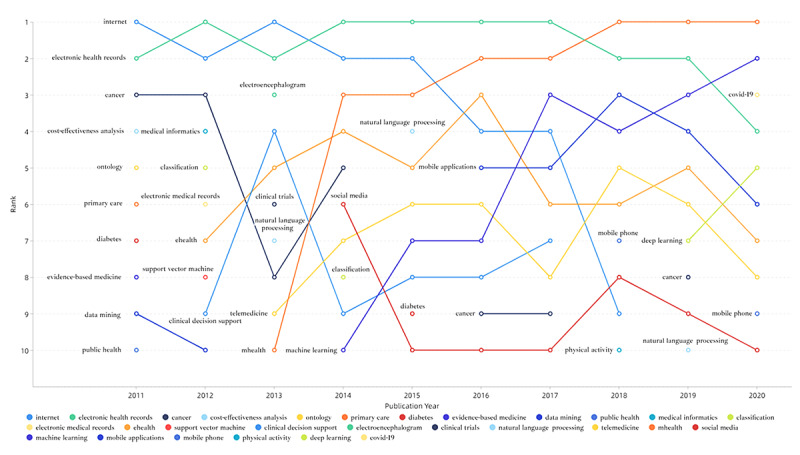
Theme evolution of the annual top 10 author keywords on medical informatics publications from 2011 to 2020.

We analyzed the theme evolution of the annual top 10 author keywords from 2011 to 2020, as shown in Figure 8. From 2011 to 2020, 28 author keywords entered the annual top 10 author keywords. The annual top 10 author keywords were constantly changing. *EHRs* was the only author keyword that has been in the annual top 10 for 10 consecutive years. *COVID-19*, which was ranked third in 2020, was the emerging theme of Medical Informatics.

## Discussion

### The Global Publication Trends of Medical Informatics

As shown in Multimedia Appendix 1, the global output of the entire life sciences and biomedical field shows a rapid growth trend from 2011 to 2020, except for 4 research areas. From 2011 to 2020, the increase rate of popularity of medical informatics far exceeded the growth rate of other research areas in life sciences and biomedical sciences, and it was almost 4 times the average increase rate, indicating the vast potential of medical informatics research in the future.

Figure 1 shows that most countries/regions have contributed to medical informatics research. Figure 2 shows that the United States was continuously ranked as the top productive country, indicating its outstanding contribution to the field of medical informatics. As the only developing and Asian country among the top 10 productive countries, China has leaped to second place in the top 10 productive countries in 2018, 2019, and 2020. The number of articles from China increased from 87 in 2011 to 946 in 2020, with a growth rate of 987.36%. China’s growth rate was much higher than the average growth rate (193.86%, from 1987 in 2011 to 5839 in 2020), enabling it to improve its ranking rapidly. The rapid development of medical informatics in China may be attributed to the fact that China’s medical reform in 2009 focused on the application of medical informatics technology. The number of articles from Germany increased from 124 in 2011 to 295 in 2020, with a growth rate of 137.90%. The growth rate of Germany was lower than the average growth rate (193.86%, from 1987 in 2011 to 5839 in 2020), which might be the main factor for its decline in ranking.

As shown in Table 1, the top 10 productive journals published 21,861 articles, accounting for 62.92% (21,861/34,742) of all medical informatics articles in the past 10 years. These journals have made substantial contributions to the development of medical informatics. From 2011 to 2020, the annual number of articles published in *Statistics in Medicine* was relatively stable, with minor fluctuations between 263 and 403. However, the ranking of *Statistics in Medicine* dropped from the first in 2011 to sixth in 2020, and its proportion dropped from 13.24% in 2011 to 5.53% in 2020. Except for 2014, the annual number of articles published by *Journal of Medical Internet Research* has continued to increase from 2011 to 2020. In particular, the annual number of articles published by *Journal of Medical Internet Research* has increased by 8.96 times, from 111 in 2011 to 1106 in 2020. *Journal of Medical Internet Research* was the most influential journal in medical informatics from 2011 to 2020 regardless of publications and citations. *IEEE Journal of Biomedical and Health Informatics* and *JMIR mHealth and uHealth*, published since 2013, were the fastest-growing journals in this field in the past 10 years.

### The Global Collaboration Patterns of Medical Informatics

The international collaboration of authorship is attributed to a fast-growing increase in the number of outputs in a certain field [29]. In the past decade, an increasing number of authors, institutions, and countries/regions contributed to the productivity of medical informatics. From 2011 to 2020, more than 110,000 authors and 20,000 institutions from 161 countries/regions published medical informatics research. Additionally, to further demonstrate the dynamic changes of the authors in the field of medical informatics, we analyzed the authors who published medical informatics articles by categorizing the last decade as 2011-2015 and 2016-2020. Among the 79,829 authors who published medical informatics articles between 2016 and 2020, only 10,051 (12.59%) authors had previously published in this area. However, 69,778 (87.41%) authors published their first medical informatics papers during this period. Thus, many new researchers have flooded into medical informatics research in the past 5 years.

The top productive author David W Bates enjoyed an international reputation in medical informatics research, focusing on medical information technology to improve the safety and quality of medical care [30,31]. As shown in Figure 3, 70 of the 304 productive authors were not in the co-authorship network, indicating that the collaboration between productive authors still had certain limitations. Harvard University was always the top productive institution and at the center of the co-authorship network of institutions, indicating its substantial academic influences in medical informatics research. As shown in Figure 4, all the 133 productive institutions were in the co-authorship network, showing extensive collaborations between institutions worldwide. The United States continuously remained the top productive country and at the center of the co-authorship network of countries/regions, indicating its substantial academic influences in medical informatics research. As shown in Figure 5, 158 of the 161 countries/regions were in the co-authorship network, showing extensive collaborations between different countries.

### The Basic Knowledge of Medical Informatics

Co-citation analysis can comprehensively demonstrate the knowledge base of a certain discipline [32]. As shown in Figure 6, the red cluster represents journals on computer science; the blue cluster represented journals on statistics science; the green cluster represented journals on medical informatics; the yellow cluster represented journals on general medicine and science and technology. In this study, co-citation analysis of cited journals showed that the knowledge base of medical informatics comes from medical informatics itself and disciplines such as computer science, general medicine, statistics, science and technology, and others.

### The Research Hotspots of Medical Informatics

The co-occurrence analysis of high-frequency author keywords clarified the leading hotspots of medical informatics research. As shown in Figure 7, there are 4 main research hotspots on medical informatics from 2011 to 2020.

The red cluster focused on AI in health care and medicine. AI usually refers to computing technology that mimics or simulates the processes supported by human intelligence, which dramatically improves diagnosis and treatment accuracy and the entire clinical treatment process [33]. With the improvement in computer performance and the availability of big data from EHRs, the research and application of AI in health care and medicine have developed rapidly. With its advanced algorithms and learning capabilities, AI applications have helped medical professionals through symptom monitoring, predictive modeling, and decision support, especially in cancer and medical imaging [34,35].

The green cluster focused on mobile health. With the popularity of the internet and the rapid development of mobile communication devices and wearable devices, mobile health has been widely used in developed and developing countries [36]. Mobile health improves the ability of health systems to provide high-quality health care, especially in chronic disease, mental health, physical activity, HIV, and smoking cessation [37-40].

The blue cluster focused on the implementation and evaluation of EHRs. EHRs utilize information systems to store a digital format for patient and population health information [41]. Quantitative or qualitative methods were applied to evaluate the usability, interoperability, security, privacy, and other functions to improve EHRs continuously [42-44]. Health care professionals can access EHRs quickly and effectively to better serve patients and the population, and these have great potential in improving medical efficiency and quality [45]. Implementing EHR and decision support helps clinicians make precise decisions to improve health care, reduce medical errors, and ensure patient safety [46-48].

The yellow cluster focused on medical informatics technology application in public health. The medical informatics technology represented by social media provides a series of possibilities for establishing multidirectional communication and interaction and quickly monitoring public emotions and activities. The application of new medical informatics technology can help increase the coverage and efficiency of public health services, especially in public communication, education, survey, engagement, and monitoring [49,50]. As our understanding of the most effective methods of using medical informatics technology to support public health research and practice matures, there will be more innovative applications of medical informatics technology in the field of public health, thereby making more remarkable contributions to improving population health.

### The Theme Evolution and Emerging Frontiers of Medical Informatics

As shown in Figure 8, the content and ranking of the top 10 author keywords have evolved dramatically every year from 2011 to 2020. Only one of the top 10 author keywords in 2011 still appeared in the top 10 keywords in 2020. These were a microcosm of the rapid development of medical informatics and show that the theme of medical informatics research was significantly changing with the development of information technology.

*EHRs* was the only author keyword that continuously ranked in the top 10 during the past 10 years, and it was the most high-frequency keyword from 2011 to 2020. EHR was the most significant research hotspot of medical informatics throughout the past decade.

The *internet* consecutively ranked second from 2011 to 2015, but its ranking showed a gradual decline after 2016, showing that internet research based on traditional computers was the most concerned research theme in the early stages of medical informatics research in the past decade.

*mHealth* first appeared in the top 10 author keywords in 2013, and its ranking increased every year. Since 2018, mHealth is ranked as the number 1 author keyword for 3 consecutive years. Moreover, the author keywords *mobile applications* ranked sixth in 2020 and *mobile phones* ranked ninth in 2020, which were closely related to mHealth, showing that mHealth based on mobile devices has become the undisputed most prominent emerging theme in medical informatics.

*Machine learning* first appeared in the top 10 authors keywords in 2014 and has remained in the top 10 author keywords since then. The main methods of AI technology, machine learning and deep learning, were ranked second and fifth, respectively, in 2020, revealing that AI in health care was an emerging frontier of medical informatics. Especially, deep learning with the ability to mine a large amount of multimodal unstructured information and the ability to automate feature learning can promote the application of data-driven solutions in disease diagnosis and predicting prognosis [51,52].

The keywords related to health care and disease such as *cancer*, *diabetes*, *physical activity*, and *COVID-19* also appeared in the top 10 author keywords, indicating that the medical informatics technology has promising applications in treating, managing, monitoring, and preventing disease in the past decade. The outbreak of COVID-19 has had an unprecedented impact on global health, economy, and society. Various active response measures have been used to deal with the epidemic, and medical information also plays an important role, especially in coordinating medical resources, information dissemination, contact tracing, public education, and mental health intervention [53,54].

### Limitations

Our research has some limitations. First, only English articles were retrieved in this study. Therefore, language bias may inevitably occur. Second, we did not evaluate the quality of publications, and some low-quality publications may have the same weight as high-quality publications. Finally, the data for this analysis were only extracted from WoSCC, excluding those from other databases such as Scopus, PubMed, or Google Scholar. Thus, publications appearing only through one of these databases may have been missed. Exploring ways to combine different data sources in future work is essential.

### Conclusions

To our knowledge, this study provided the first comprehensive picture of global efforts on medical informatics in the past decade from a bibliometric analysis perspective. We summarize the recent advances in medical informatics in the past decade and shed light on their publication trends, influential journals, global collaboration patterns, basic knowledge, research hotspots, theme evolution, and emerging frontiers. These findings will accurately and quickly grasp the research trends and provide valuable guidance for future medical informatics research.

## Appendix

Multimedia Appendix 1 The global output of the entire life sciences and biomedical field.

## Abbreviations

AI: artificial intelligence
EHRs: electronic health records
WoSCC: Web of Science Core Collection
